# Transient Hyperphosphatasemia Following Pediatric Kidney Transplant

**DOI:** 10.7759/cureus.17697

**Published:** 2021-09-03

**Authors:** Elisabeth B Cole, Melissa Anslow, Paul Fadakar, Yosuke Miyashita, Armando Ganoza, Michael L Moritz

**Affiliations:** 1 Pediatric Nephrology, University of Pittsburgh Medical Center (UPMC) Children's Hospital of Pittsburgh, Pittsburgh, USA; 2 Hillman Center for Pediatric Liver Transplantation, University of Pittsburgh Medical Center (UPMC) Children's Hospital of Pittsburgh, Pittsburgh, USA

**Keywords:** pre-pubertal post-kidney transplant patients, elevated alkaline phosphatase (alp), transient hyperphosphatasemia (th), alkaline phosphatase, pediatric kidney transplant

## Abstract

Introduction

Transient hyperphosphatasemia (TH) is a rare benign condition of elevated serum alkaline phosphatase (AP) levels seen in healthy children. TH has been reported to occur in pediatric solid organ transplants, including kidney transplant patients. Little is known about TH in pediatric kidney transplant patients.

Objective

To evaluate the incidence and natural history of TH in pediatric kidney transplant patients.

Methods

A retrospective chart review of patients < 18 years of age who underwent kidney transplantation at the University of Pittsburgh Medical Center (UPMC) Children's Hospital of Pittsburgh between 2008 and 2019 was performed to identify patients with TH, defined as an AP level greater than 1,000 IU/L. Exclusion criteria included repeat kidney transplants or kidney transplant as part of a multiorgan transplant.

Results

One hundred seventy-six patients underwent a solitary kidney transplant, of which 87 were less than 12 years of age. Eleven patients (6.5%) were found to have TH, all of whom were < 12 years of age (12.8%) (median age: 5 years; range: 1 - 11 years). The median AP level prior to transplant was 183 IU/L (range: 104 - 309 IU/L) and the median peak AP was > 2,300 IU/L (range: 1,227 - 4,912 IU/L). The median time from a kidney transplant to the diagnosis of TH was 0.6 years (range: 0.3 to 7.7 years). The median length of time that TH persisted was 0.5 years (range: 0.2 to 0.9 years). The median estimated glomerular filtration rate (GFR) at the time of diagnosis of TH was 84 mL/min/1.73m^2^ per the bedside Schwartz equation (range: 45 to 152 mL/min/1.73m^2^). One patient had variable AP levels over nine months prior to resolution; the other 10 patients had a solitary peak of AP prior to resolution. No patient required treatment of elevated AP levels and the TH resolved spontaneously without intervention. No patients had significant abnormalities of markers of metabolic bone disease or were on active vitamin D, calcium, or phosphorus supplements. Two patients reported bone pain, and one patient was found to have avascular necrosis of the hip.

Conclusions

TH is a relatively common finding following a pediatric kidney transplant in pre-pubertal children less than 12 years of age. It primarily occurs in the first year following a kidney transplant and usually resolves without recurrence within one year of onset.

## Introduction

Transient hyperphosphatasemia (TH) is a rare, benign condition of severely elevated serum alkaline phosphatase (AP) level seen in healthy children. The condition was first described by Bach in 1954 [1], and further characterized by Posen et al. in 1977 [2]. Historically, TH was described in children less than five years of age who experienced a transient increase in serum AP levels in the absence of bone or liver disease and lasting four months or less. More recently, the condition has been identified in adults, in addition to infants and children, and has been reported to occur in a wide variety of populations, including solid organ transplant patients [3-13]. The incidence of TH in the general population has been estimated to be between 1% and 3%, and studies have found the incidence of TH to be anywhere from 2% to as high as 6% in a population of liver transplant patients at a large academic medical center [3, 14-15]. The incidence of TH in the pediatric kidney transplant population is unknown. The aim of this study was to determine the incidence and describe the natural history of TH in a pediatric population of initial solitary kidney transplant recipients at a large academic medical center.

This article was previously presented as a meeting abstract during Phase I of the Pediatric Academic Societies 2021 Virtual Meeting from April 30 through May 4, 2021. 

## Materials and methods

Between 2008 and 2019, 176 pediatric patients underwent initial solitary kidney transplant at the University of Pittsburgh Medical Center (UPMC) Children’s Hospital of Pittsburgh. Of these, 87 patients were less than 12 years of age at the time of transplant. Exclusion criteria included patients who underwent a multi-organ transplant at the time of initial kidney transplant and uncontrolled secondary hyperparathyroidism prior to transplant. A retrospective chart review of this population was conducted with University of Pittsburgh Institutional Review Board (#STUDY20050403) approval to identify patients with TH, defined as a transient AP greater than 1,000 IU/L. Demographic characteristics, along with laboratory, radiographic, and clinical data from pre- and post-kidney transplant at the time of TH, were abstracted and analyzed. 

## Results

Eighteen out of 176 patients were found to have AP levels > 1,000 IU/L. Seven patients were excluded from the study after the in-depth record review; four patients did not have sufficient data in the electronic medical record to confirm the diagnosis of TH, and three patients had uncontrolled secondary hyperparathyroidism and elevated AP levels prior to transplant. Of those excluded, one was less than 12 years of age at the time of transplant. Of the transplant population as a whole, 11 out of 169 patients were identified as having TH (6.5%), and of those patients less than 12 years of age at the time of transplant, 11 out of 86 patients were identified as having TH (12.8%). 

**Figure 1:**
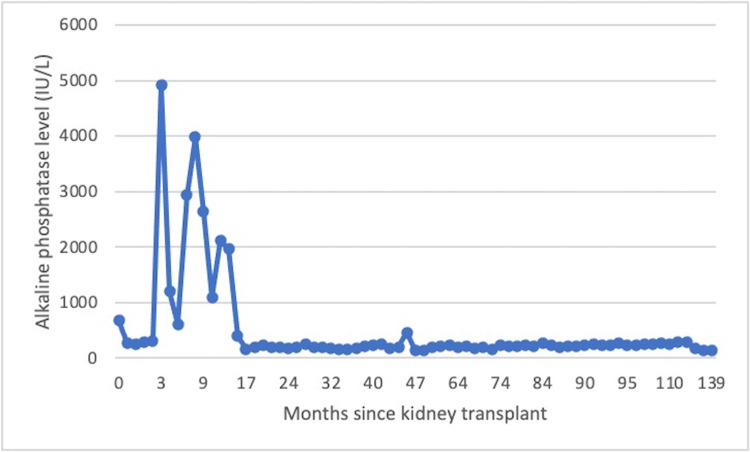
Trend of alkaline phosphatase levels for Patient 4. Levels fluctuated over the course of nine months, at one point dropping below 1,000 IU/L before rising again, prior to ultimate resolution.

Characteristics of the patients with TH are listed in Table 1. Four patients were female (35%). The causes of end-stage kidney disease (ESKD) were varied, including congenital anomalies of the kidney and urinary tract, medication side effects, glomerular disease, and unknown. All patients had Stage V chronic kidney disease prior to transplant; six patients had received at least one treatment of dialysis prior to transplant. The median age at kidney transplant was five years (range: 1 to 11 years), and all patients were less than 12 years of age at the time of kidney transplant. The median AP level across included patients immediately prior to transplant was 183 IU/L (range: 104-309 IU/L). Immunosuppression for transplant consisted of induction with alemtuzumab in all patients, followed by tacrolimus alone (three patients), or in combination with either CellCept (seven patients) or Imuran (one patient); no patients were on daily prednisone. The median time from a kidney transplant to the diagnosis of TH was 0.6 years (range: 0.3 to 7.7 years). The median estimated GFR at the time of diagnosis of TH was 84 mL/min/1.73m^2^ per the Schwartz equation (range: 45 to 152 mL/min/1.73m^2^). No patients included in the study had delayed graft function. The median peak AP was > 2,300 IU/L (range: 1,227 - 4,912 IU/L) (Table 1). The median length of time that TH persisted was 0.5 years (range: 0.2 to 0.9 years). One patient (Patient 4) had fluctuating AP levels between 610 IU/L and 4,912 IU/L over the course of 0.75 years (nine months) prior to the ultimate resolution of TH (Figure 1). The other 10 patients had a solitary peak of AP prior to resolution. No additional testing or treatment were undertaken for any patient with TH. 

**Table 1:** Demographic Characteristics and Study Findings of Included Patients AP: alkaline phosphatase; CKD: chronic kidney disease; CNI: calcineurin inhibitor; ESRD: end-stage renal disease; FSGS: focal segmental glomerulosclerosis; GFR: glomerular filtration rate; MCKD: multicystic dysplastic kidney; PUV: posterior urethral valves; RVT: renal vein thrombosis; Stage Vd: Stage V on dialysis; TH: transient hyperphosphatemia; unk: unknown; UPJ: ureteropelvic junction; VACTERL: vertebral defects, anal atresia, cardiac defects, tracheoesophageal fistula, renal anomalies, limb abnormalities

Subject	Sex	Cause of ESRD	Stage of CKD prior to transplant	Time on dialysis pre-transplant, if applicable	Age at kidney transplant (years)	AP level prior to transplant (IU/L)	Time from transplant to TH (years)	Peak AP level (IU/L)	Estimated GFR (mL/min/1.73m^2)	Time from peak AP level to resolution of TH (years)
1	Male	VACTERL	Stage V		11	234	2.9	2841	45	0.17
2	Male	PUV	Stage Vd	22 months	3	309	7.7	1227	48	0.17
3	Male	Bilateral RVT	Stage Vd	One treatment	5	181	0.3	2482	152	0.17
4	Female	Bilateral UPJ obstruction	Stage Vd	29 months	3	unk	0.3	4912	65	0.75
5	Male	Cystic renal dysplasia	Stage V		1	104	1.7	2395	97	0.17
6	Male	Right ectopic MCDK, left hypoplastic kidney	Stage V		7	155	0.5	1692	130	0.17
7	Female	Unknown etiology	Stage Vd	10 months	4	185	0.4	>2330	150	0.92
8	Female	FSGS and reflux nephropathy	Stage Vd	2 months	10	197	1.4	1528	108	0.50
9	Male	CNI toxicity	Stage Vd	1 month	7	306	1.3	>2330	81	0.67
10	Male	FSGS	Stage V		5	147	0.6	>2330	83	0.67
11	Female	FSGS	Stage V		10	164	0.4	>2330	84	0.58

No patient had significant abnormalities of serum calcium, phosphorus, 25-OH vitamin D, or parathyroid hormone levels at or around the time of presentation with TH. No patient had an elevation of aspartate aminotransferase or alanine transaminase immediately prior to, during, or following TH. During the study period, two patients reported bone pain; both resolved while the AP level remained elevated. Two patients had bone x-rays; one showed avascular necrosis of the hip, and the other showed delayed bone age. Four patients had upper respiratory tract symptoms either before or at the time of elevated AP levels. Three patients had Epstein-Barr virus (EBV) viremia, and one had a respiratory syncytial virus (RSV) during the study period; the time course of these viral infections did not correlate with TH. Four patients had a diarrheal illness prior to or at the time of the onset of an elevated AP level; two were attributed to medication side effects, one was attributed to post-transplant lymphoproliferative disorder, and one cause was unknown. Two patients were on cholecalciferol at the time of TH. No patient was on calcitriol, calcium, or phosphorus-containing medications, or prednisone. Seven of the 11 patients were on Bactrim™ (trimethoprim and sulfamethoxazole) for urinary tract infection prophylaxis and/or pneumocystis prophylaxis. 

## Discussion

There have been multiple case reports of TH in solid organ transplant recipients, including kidney transplant patients; yet, the incidence of TH in this specialized population has not been described [4, 6, 9-10, 13]. In this study, the overall incidence of TH at a large academic medical center was 6.5%, similar to the incidence found in other solid organ transplant recipients and higher than the incidence of TH that has been previously described in healthy children [15-16]. All of the patients diagnosed with TH were less than 12 years of age, resulting in a relatively high incidence of 12.8% for transplant recipients less than 12 years of age. This is consistent with the epidemiology of TH in healthy children, which is primarily a condition of pre-pubertal children [17]. 

While routine monitoring every three to six months was the benchmark, AP levels in this cohort were not routinely measured in the post-transplant period as part of bone health. Rather, the AP level (as a component of liver function tests) was checked periodically at the discretion of the provider or when clinically indicated. As such, TH was an incidental finding in all patients in this study. This study likely underestimates the true incidence of TH, as there were likely additional patients who experienced transient elevations in AP levels that were clinically insignificant and were unidentified due to lack of AP testing. The majority of TH episodes developed within one to two years following transplant, though TH was seen as long as seven years after transplant in one patient. In addition, TH resolved within one year of the onset of elevated AP levels in most cases. Only one patient in the study had variable fluctuations in AP levels prior to ultimate resolution (Figure 1). This further supports the transient nature of TH, with quick onset and resolution without any clinical implications. All patients in this study had resolution of TH without intervention, as has been found in prior studies, and no subject had signs or symptoms of clinically significant hyperphosphatasemia. There was no identifiable trend in infectious history or medication administration to explain TH seen in the patients in this study. 

The main limitation of this study was the retrospective nature of the data collection which resulted in limited or missing data. As AP levels were not routinely followed in the post-transplant population, the time to resolution of TH may be overestimated as the level may have resolved before the next set of labs were obtained, and the overall incidence may be underestimated, as stated above. No patients had an evaluation of the isoforms of AP completed, which limits the ability to interpret the elevated AP level. Assessment of isoforms of AP could be considered an unnecessary evaluation for TH and would likely have been considered in patients with persistent hyperphosphatasemia.

## Conclusions

This is the first pediatric study to elucidate the incidence and natural history of TH in post-kidney transplant patients. Understanding the time frame within which TH can be seen, the extent to which AP can be elevated in TH, the length of time TH persists, and the lack of clinical ramifications of elevated AP levels is important for avoiding invasive testing, unnecessary treatment, and undo stress for the patient and their family in the setting of an uncertain diagnosis. This study demonstrates that TH can be seen following kidney transplant, is relatively common in pre-pubertal kidney transplant patients and resolves without intervention generally within one year of onset. TH does not require further evaluation with costly or invasive testing, even in the specialized population of post-kidney transplant patients. 
